# RNA binding proteins involved in regulation of protein synthesis to initiate biogenesis of secondary tumor in hepatocellular carcinoma in mice

**DOI:** 10.7717/peerj.8680

**Published:** 2020-03-20

**Authors:** Genliang Li, Anni Ni, Yulian Tang, Shubo Li, Lingzhang Meng

**Affiliations:** Department of Biochemistry and Molecular Biology, Youjiang Medical University for Nationalities, Baise, Guangxi, China

**Keywords:** RNA binding proteins, Gene expression regulation, Tumor microenvironment, Protein synthesis, Biogenesis of secondary tumor, Hepatocellular carcinoma

## Abstract

**Background:**

The tumor microenvironment (TM) in close contact with cancer cells is highly related to tumor growth and cancer metastasis. This study is to explore the biogenesis mechanism of a secondary hepatocellular carcinoma (HCC) based on the function of RNA binding proteins (RBPs)-encoding genes in the physiological microenvironment (PM).

**Methods:**

The healthy and HCC mice were used to isolate the PM, pre-tumor microenvironment (PTM), and TM. The samples were analyzed using the technology of RNA-seq and bioinformatics. The differentially expressed RBPs-encoding genes (DERs) and differentially expressed DERs-associated genes (DEDs) were screened to undergo GO and KEGG analysis.

**Results:**

18 DERs and DEDs were identified in the PTM vs. PM, 87 in the TM vs. PTM, and 87 in the TM vs. PM. Those DERs and DEDs participated in the regulation of gene expression at the levels of chromatin conformation, gene activation and silencing, splicing and degradation of mRNA, biogenesis of piRNA and miRNA, ribosome assemble, and translation of proteins.

**Conclusion:**

The genes encoding RBPs and the relevant genes are involved in the transformation from PM to PTM, then constructing the TM by regulating protein synthesis. This regulation included whole process of biological genetic information transmission from chromatin conformation to gene activation and silencing to mRNA splicing to ribosome assemble to translation of proteins and degradation of mRNA. The abnormality of those functions in the organic microenvironments promoted the metastasis of HCC and initiated the biogenesis of a secondary HCC in a PM when the PM encountered the invasion of cancer cells.

## Introduction

Malignant tumor, as a new “system” in the body, is gestated by the organic environment which is the time and space for a tumor system to survive and to proliferate, especially the microenvironment that is in close contact with cancer cells. The latter is the tumor microenvironment (TM) (Shirako et al., 2015; Gouirand, Guillaumond & Vasseur, 2018). Tumorigenesis is a complex pathological process which involves the integral TM but not certain cells. Therefore, research from the entire TM rather than specific cells may be more helpful to better our understanding of the mechanism of tumorigenesis. Both tumor growth and cancer metastasis are closely related to various stromal cells and their secretory proteins including chemokines in the TM (Shirako et al., 2015; Gouirand, Guillaumond & Vasseur, 2018). RNA and its binding proteins are also important components in the cancer (Jiang & Baltimore, 2016; Brody & Dixon, 2018). The RNAs play important roles in the pathological and physiological tissues including cancer. For example, a large number of non-coding RNAs (ncRNA), such as miRNA, piRNA, circRNA, etc., are involved in the regulation of gene expression and cell function (Dehghani Amirabad et al., 2018). The functions of those RNAs is affected and regulated by RNA binding proteins (RBPs) (Dehghani Amirabad et al., 2018; Ding et al., 2018; Dong et al., 2019). The RBPs are related with the tumorigenesis and cancer metastasis (Jeng et al., 2008; Hopkins et al., 2016; Dang et al., 2017). However, it is still enigmatic about the exact processes in which RBPs-encoding genes and the relevant genes participate in transcriptome level during the biogenesis of secondary tumor in the activity of hepatocellular carcinoma (HCC) cells. Due to extensive functions of diversity RNAs from protein synthesis to multi-level regulation of gene expression, we proposed a hypothesis that RBPs were involved in the biogenesis of secondary tumor through binding diversity RNAs to affect those functions of them in a physiological microenvironment (PM) for transformation of pre-tumor microenvironment (PTM) to construct a TM (Shirako et al., 2015; Peinado et al., 2017).

Therefore, in our study, we adopted a murine HCC model, isolated healthy liver tissue as a PM, the tumor tissue after cancerization as a TM, and the non-cancerous liver tissue after tumor formation as a PTM. The expressions and functions of RBPs-encoding genes and RBPs-associated genes in the PM, PTM, and TM were analyzed using the technology of RNA-seq and bioinformatics to explore the biogenesis mechanism of a new tumor in the PM based on the functions of those genes. Although HCC may metastasize to a variety of tissues, liver tissue and HCC tissue have more similarities in source. Therefore, we choose liver tissue instead of other tissues as PTM in this study.

## Materials & Methods

### The data of RNA-seq

The RNA-seq data come from previous sequencing results in our laboratory. There were totaling 89.32, 86.20, and 89.68 million bases for PTM, PM, and TM, respectively, respectively, to be used in this analysis. The data of RNA-seq were deposited in the SRA database in NCBI, accession numbers are SRR8156139, SRR8156138, SRR8156137, SRR8157215, SRR8157216, and SRR8157217.

The construction of liver cancer mouse model, RNA extraction, and sequencing were showed in our previous study (Li et al., 2019c). Briefly, at 7 weeks of age, healthy Kunming mice weighing 22–25 g were purchased from the Experimental Animal Center in Youjiang Medical University for Nationalities. 150 mice were randomly and equally divided into two groups. The H22 cell line purchased from Kaiji Biotechnology Co., Ltd. (Nanjing, China) were subcutaneously injected into double forelimbs of mice in one group at a concentration of 10^5^ cells/ml. Each mouse was injected about 0.2 ml H22 cell suspension. Another group were injected using physiological saline replacing H22 cells as a control. After 4 weeks of regular feeding, the tumors which occurred at the inoculation sites but not in the livers in the 40 mice were dissected as TM samples. At the same time, their livers in which there was not tumor were dissected as PTM ones. The healthy mouse livers in the control were used as PM samples. The care of all animals complied with experimental animal ethics guidelines of Youjiang Medical University for Nationalities (the approval reference number: SCXK Gui 2017-0003). The RNA extraction kit (Invitrogen, Shanghai, China) was used to extract total RNA according to the instructions. Illumina HiSeq 2,500 were used for RNA to sequence the mixture of all total RNA from 40 samples in same group with paired-end reads (2 ×150 bp) referring to our previous study (Li et al., 2019c). RNA-seq of each sample was repeated twice. RNA quality detection, reverse transcription, library construction, RNA-seq and sequencing data processing referred to our previous method (Li & Qian, 2017).

**Figure 1:**
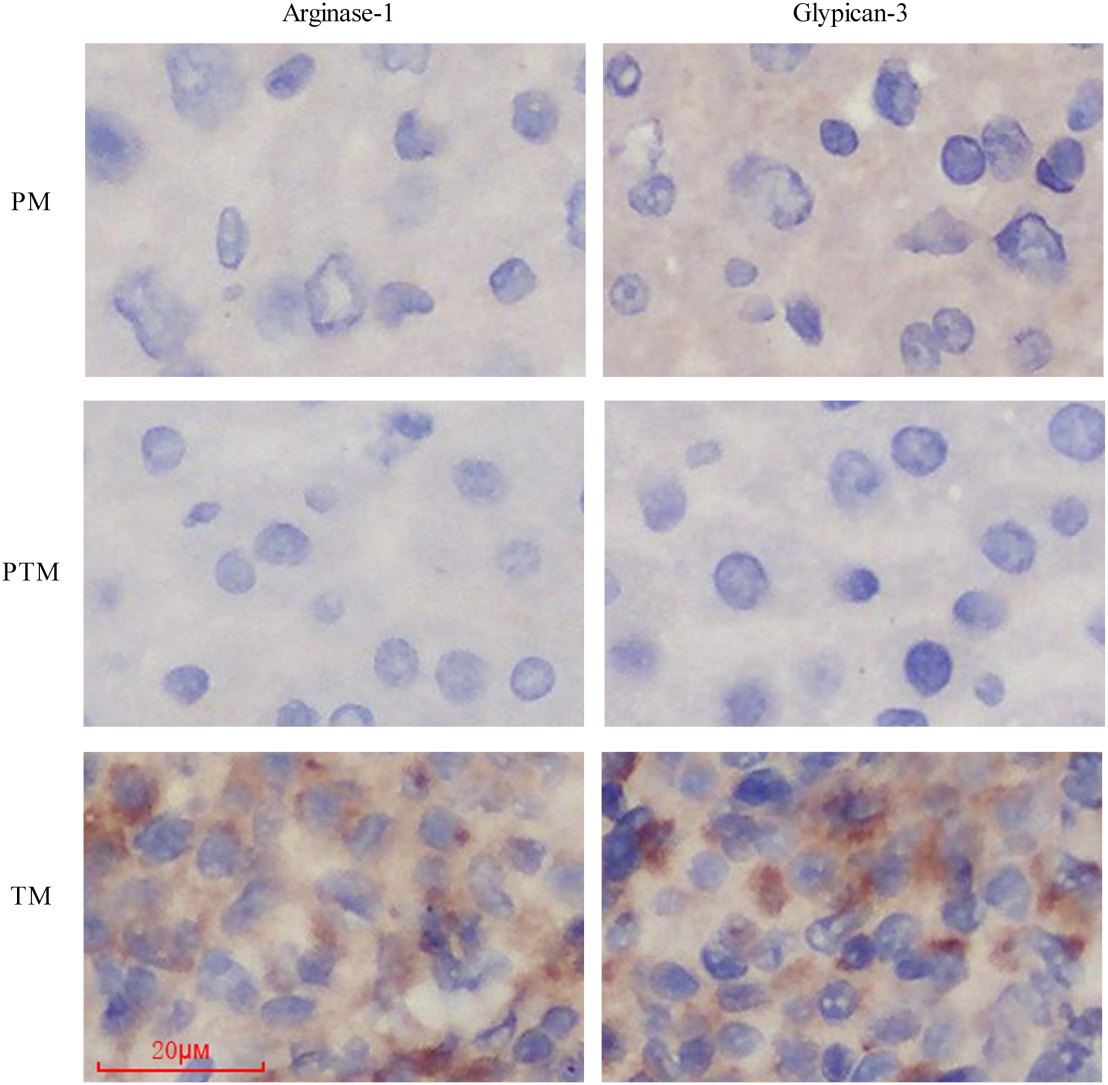
The expression of two cancer maker proteins, Arginase-1 and Glypican-3 in these different tissues using immunostaining.

To confirm the PM, the TM and the PTM used in this study are right, we investigated expression of two cancer maker proteins, Arginase-1 and Glypican-3 in these different tissues using immunostaining. Briefly, samples were collected, fixed by formalin and embedded by paraffin. Immunostaining was performed using the immune peroxidase method, referring to Obiorah et al. (2019). Pretreated 4-*μ*m-thick sections from the tissue blocks were incubated with monoclonal Glypican-3 (GPC-3) antibody and monoclonal Arginase-1 (ARG-1) antibody, purchased from Maxim (Fuzhou, China), respectively. The immunostaining results showed that the TM tissues could be labeled by both markers, Arginase-1 and Glypican-3, while both PM and PTM tissues were not (Fig. 1). The results can confirm the PM, the TM and the PTM used in this study are right.

### GO and KEGG analysis of differentially expressed RBPs-encoding genes and relevant genes

The mouse genomics (http://www.ncbi.nlm.nih.gov/genome/?term=Mus%20musculus%20%5BOrganism%5D) were previously used to map the RNA-seq and then the differentially expressed genes (DEGs) were statistically analyzed (Li et al., 2019c). In this study, we downloaded RBP genes and RBP-associated genes from the nucleotide database in NCBI and STRING web to construct the blast genetic library. The false discovery rate (FDR) and the probability of differentially expressed RBPs-encoding genes (DERs) and differentially expressed DERs-associated genes (DEDs) were set at *q* <0.05 and *p* <0.05, respectively. The DERs-associated genes referred those which can be intertwined with DERs in the interaction using the Version 11.0 STRING web (https://string-db.org/) online analysis. The DEGs from the RNA-seq data were then mapped into the library to picked out DERs and DEDs. The enrichment analysis of Gene Ontology (GO) terms and the Kyoto Encyclopedia of Genes and Genomes (KEGG) were online performed using DAVID 6.7 software (https://david.ncifcrf.gov/).

### Interaction analysis of DERs- and DEDs-encoded proteins

The interaction of Co-expression (or Text-mining if no Co-expression) analysis of DERs- and DEDs-encoded proteins were online performed using Version 11.0 STRING web.

### Analysis of AS and SNV/INDEL

To explore the causes of DERs and DEDs abnormal expression, we analyzed the alternative splicing (AS) of those DEGs using for classification and expression statistic by ASprofile (V1.0.4) software. The mpileup process was performed using samtools (v0.1.18) software to obtain possible SNV/INDEL results which then were annotated with annovar (v2013.02.11) software for each sample.

### Validation of DEGs using RT-qPCR

The DEGs involving the tumor biology were picked out from DERs and DEDs for quantitation in the samples to validate the RNA-seq results. The real-time quantitative polymerase chain reaction (RT-qPCR) were used for the quantitation according to the manufacturer’s instructions. The experiment was repeated three times. The conditions of RT-qPCR reactions, designment of the primers and analysis of RT-qPCR in the study referred to our previous method (Li et al., 2019c). The primers were synthesized by Sangon Biotech (Shanghai, China) (Table 1).

**Table 1:** The primers of RT-qPCR.

Gene	5′-primer	3′-primer
RN18s	AGGGGAGAGCGGGTAAGAGA	GGACAGGACTAGGCGGAACA
FOXA3	CTACATGACCTTGAACCCACTC	GGGCTACATACCCGGAAGC
CTNNA1	AAGTCTGGAGATTAGGACTCTGG	ACGGCCTCTCTTTTTATTAGACG
AGT	TCTCCTTTACCACAACAAGAGCA	CTTCTCATTCACAGGGGAGGT
HGF	AAAGGGACGGTATCCATCACT	GCGATAGCTCGAAGGCAAAAAG
MAFA	AGGAGGAGGTCATCCGACTG	CTTCTCGCTCTCCAGAATGTG

## Results

### DERs and DEDs

There were 33 DERs among 58 RBPs-encoding genes found in this study in all samples (table s1). Those DERs were intertwined with 87 genes in the interaction using the Version 11.0 STRING web. Among them, there were 72 DEDs. The mean fold changes of DERs or DEDs were larger than 2 between the PTM and the PM, the TM and the PTM, or the TM and the PM. Those differentially expressed genes (DEGs) included 2 types, upregulated and downregulated.

In the TM vs. the PM (TM/PM), there were 27 DERs, among which expression of 19 were upregulated and expression of 8 were downregulated. The former included KHDRBS2, ELAVL4, CELF6, ADAD1, TRA2B, LIN28A, and so on. The latter included MALAT1, CSDC2, RBFOX3, IGF2BP3, TARBP1, RBM47, and so on. As for DEDs, we found 60 in our sequencing data, among which there were 48 with expression upregulation and 12 with expression downregulation. The former included PIWIL1, NANOG, PVALB, SNRPB, LSM6, MYC, and so on. The latter included CMBL, CD81, SEPP1, CDH1, MYCN, ESRP2, and so on.

In the PTM vs. the PM (PTM/PM), only 4 DERs were present and their expressions all were upregulated. They were RBFOX3, IGF2BP3, ELAVL2, and LIN28A. Our sequencing results also showed that there were 14 DEDs pertaining to those 4 DERs, including 12 with expression upregulation and 2 with expression downregulation. The former included PVALB, GFAP, PIWIL2, PUS3, DCX, TDRD12, and so on, and the latter included CMBL and SYP.

As for in the TM vs. the PTM (TM/PTM), there were 23 DERs, including 17 with expression up-regulation and 6 with expression down-regulation. The genes with expression up-regulation included KHDRBS2, ELAVL4, CELF6, ADAD1, CELF5, ELAVL3, CELF4, SRP14, CD151, VMA21, TAF15, NIFK, YBX3, STAU1, ZFR, HNRNPA1, LSM4 and ones with expression down-regulation included MALAT1, LIN28A, CSDC2, RBFOX3, ifitm1, TDRKH. In our sequencing results, a total of 64 DEDs were found to be intertwined with those 23 DERs, including 54 with expressions up-regulation and 10 with expression down-regulation. The former included PVALB, SNRPB, PIWIL4, LIN28B, NANOG, IGF2BP1, and so on, and the latter included PUS3, TDRD12, SEPP1, CD81, GFAP, HGF.

**Figure 2:**
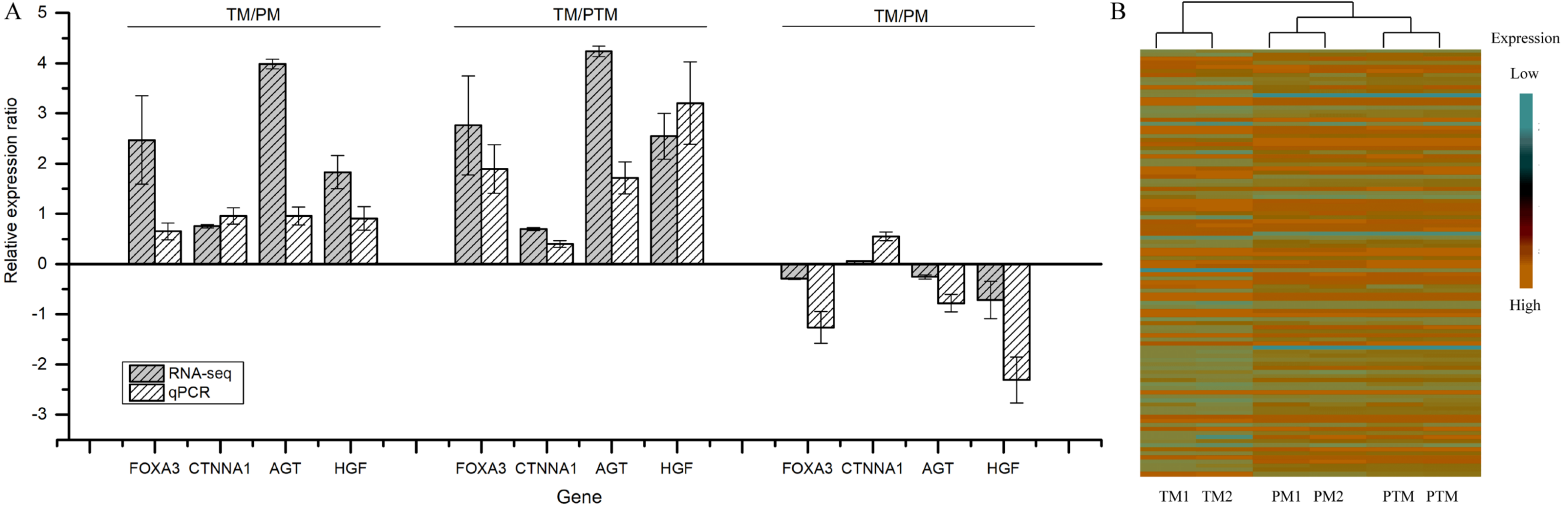
The expression levels of DEGs between the PTM, PM, and TM. (A) RT-qPCR results comparing with RNS-seq results; (B) heatmap of the DERs and DEDs in TM/PM, in TM/PTM, and in PTM/PM.

The RT-qPCR results showed that the expression levels of DERs and DEDs between the PTM, PM, and TM were consistent with those in the RNA-seq data (Fig. 2A). For example, the results obtained using two methods all showed that the 3 DEGs among 4 were upregulated except for downregulated CTNNA1 in the PTM/PM and 4 were all downregulated in both the TM/PTM and the TM/PM. The data of RT-qPCR were analyzed using the △△Ct method with RN18s as reference gene. The heatmap of DERs and DEDs indicated that the expression of those differentially expressed genes were identical in our replicas (Fig. 2B).

**Figure 3:**
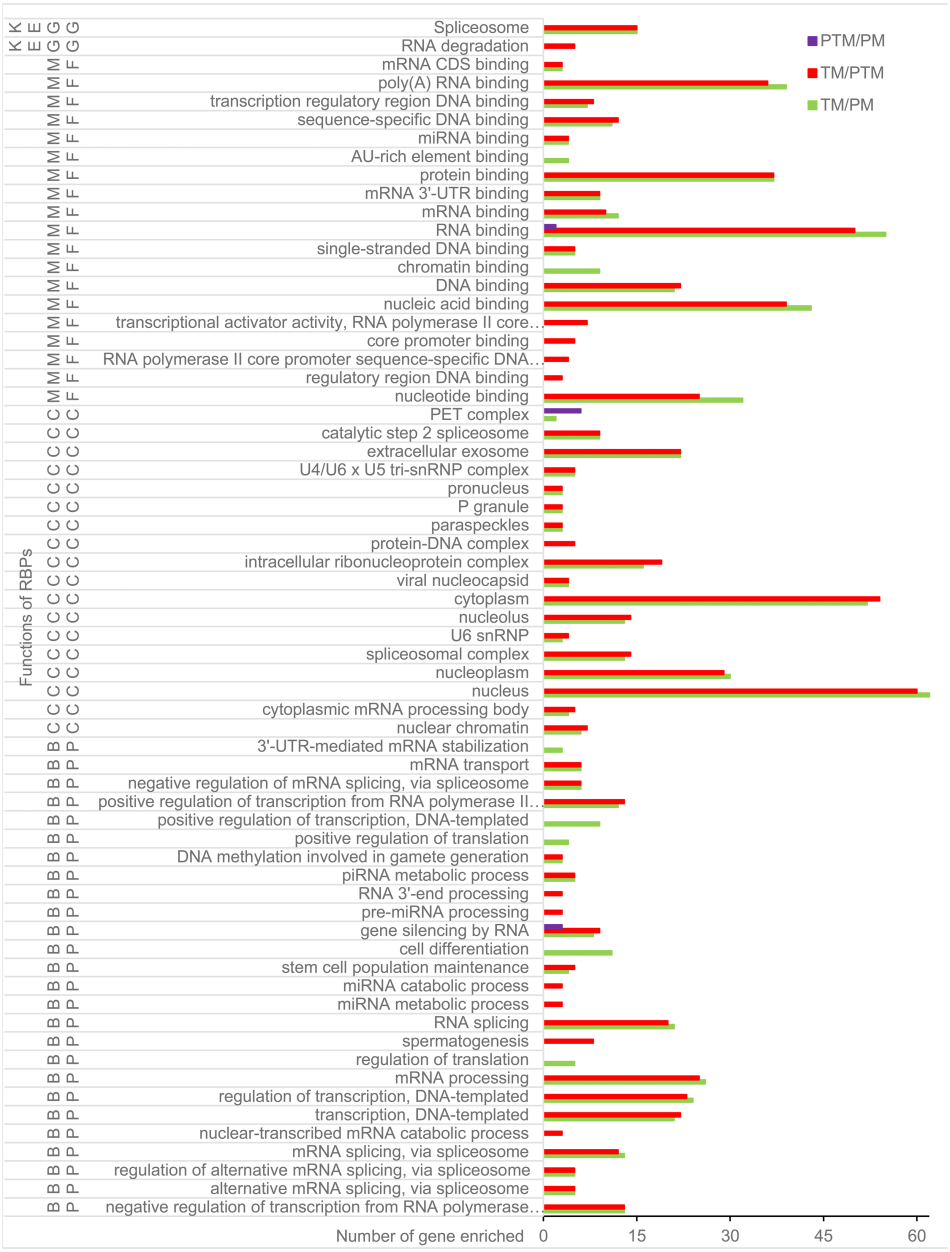
GO term and KEGG pathway of DERs and DEDs in TM/PM, in TM/PTM, and in PTM/PM. BP, biological process; CC, cellular component; DERs, differentially expressed RNA binding protein genes; DEDs, differentially expressed genes relevant to DERs; GO, Gene Ontology; KEGG, Kyoto Encyclopedia of Genes and Genomes; MF, molecular function; PM, physiological microenvironment; TM, tumor microenvironment.

**Figure 4:**
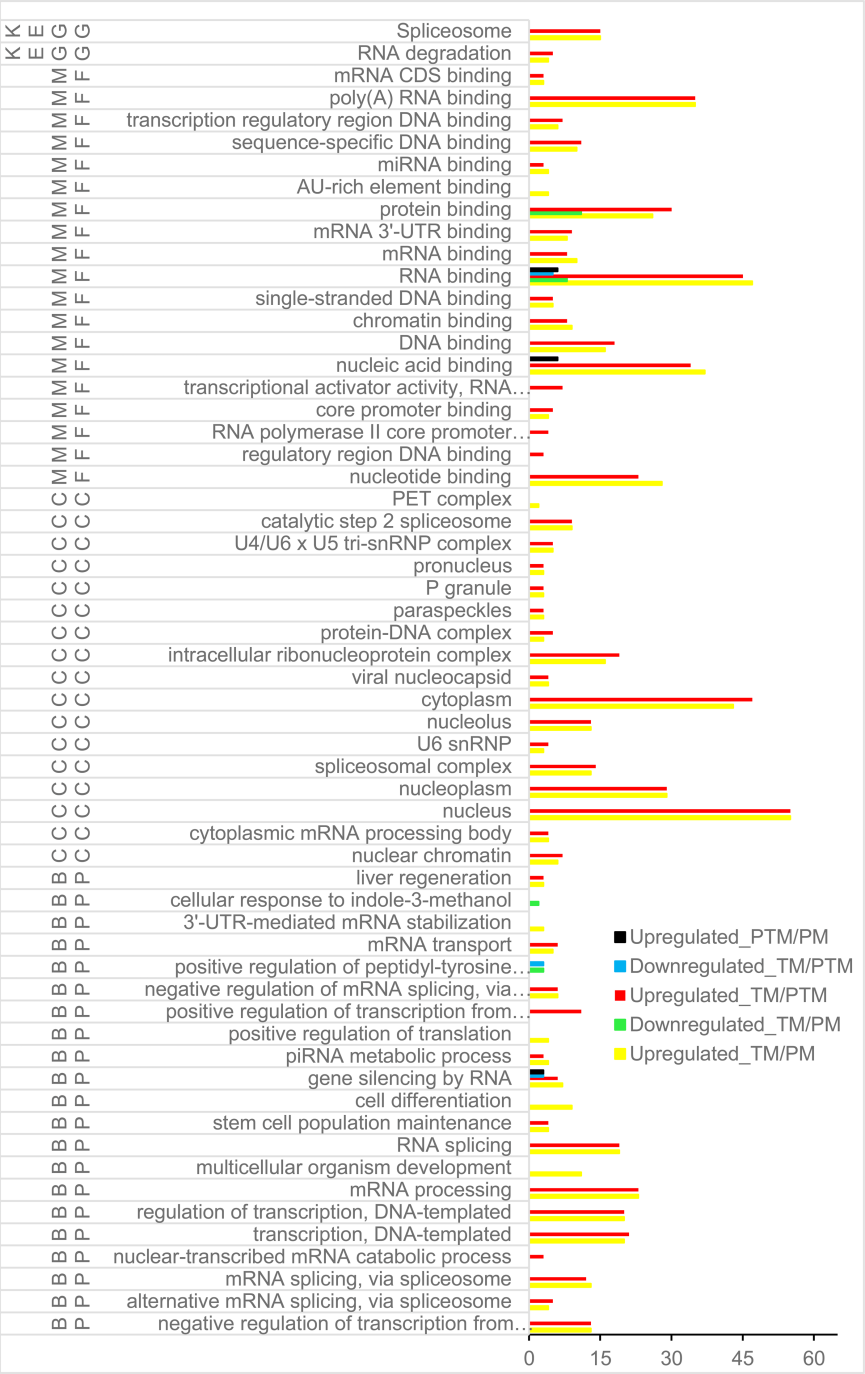
GO term and KEGG pathway of DERs and DEDs with expression upregulation in TM/PTM and in TM/PM. BP, biological process; CC, cellular component; DERs, differentially expressed RNA binding protein genes; DEDs, differentially expressed genes relevant to DERs; GO, Gene Ontology; KEGG, Kyoto Encyclopedia of Genes and Genomes; MF, molecular function; PM, physiological microenvironment; TM, tumor microenvironment.

### The functions of DERs and DEDs

The results of GO and KEGG analysis showed that the 67 DERs and DEDs with expressions up-regulation in TM/PM were enriched into 17 biological processes (BPs), 17 cellular components (CCs), and 19 molecular functions (MFs) of GO terms and 2 KEGG pathways (Fig. 3). The 20 DERs with expression down-regulation in TM/PM were enriched into 2 BPs and 2 MFs of GO terms (Fig. 4). They were not enriched into the CC and KEGG pathway. At the level of DNA and chromatin, those DEGs mentioned above were enriched into 5 MFs, such as GO:0003677 ∼DNA binding (16 genes), GO:0003682 ∼chromatin binding (9), GO:0043565 ∼sequence-specific DNA binding (10); and 2 CCs, GO:0000790 nuclear chromatin (6) and GO:0032993 protein-DNA complex (3). At the level of transcription, there were 2 BPs, GO:0006355 ∼regulation of transcription, DNA-templated (20) and GO:0000122 ∼negative regulation of transcription from RNA polymerase II promoter (13). Especially, the RNA spicing enriched 4 BPs, 2 CCs, and 1 KEGG pathway. With respect to ncRNA, those DEGs were enriched into 2 BPs, GO:0031047 ∼gene silencing by RNA (7) and GO:0034587 ∼piRNA metabolic process (4); 3 CCs, GO:0046540 ∼U4/U6 x U5 tri-snRNP complex (5), GO:0005688 ∼U6 snRNP (3), and GO:1990923 ∼PET complex (2); and 1 MF, GO:0035198 ∼miRNA binding (4). Additionally, at the level of translation, the DEGs were enriched into the GO terms GO:0044822 ∼poly(A) RNA binding (35), GO:0030529 ∼intracellular ribonucleoprotein complex (16), GO:0045727 ∼positive regulation of translation (4), mmu03018: RNA degradation (4), and so on.

By GO and KEGG analysis, the DERs and DEDs in PTM/PM only were enriched into 3 GO terms, GO:0031047 gene silencing by RNA (3 genes), GO:0003723 RNA binding (6), and GO:0003676 nucleic acid binding (6) (Fig. 4).

Among the genes with expression up-regulation in TM/PTM, the DERs together with the DEDs for GO and KEGG analysis were enriched into 13 GO terms, including 15 BPs, 18 MFs, 16 CCs, and 2 KEGG pathways (Fig. 4). The top 5 BPs included GO:0006397 mRNA processing (23 genes), GO:0006351 transcription, DNA-templated (21), GO:0006355 regulation of transcription, DNA-templated (20), GO:0008380 RNA splicing (19), and GO:0000122 negative regulation of transcription from RNA polymerase II promoter (13). The top 5 CCs included GO:0005634 nucleus (55), GO:0005737 cytoplasm (47), GO:0005654 nucleoplasm (29), GO:0030529 intracellular ribonucleoprotein complex (19), and GO:0005681 spliceosomal complex (14). The top 5 MFs included GO:0003723 RNA binding (47), GO:0044822 poly(A) RNA binding (35), GO:0003676 nucleic acid binding (34), GO:0005515 protein binding (30), and GO:0000166 nucleotide binding (23). The KEGG pathways only included mmu03040 spliceosome (15) and mmu03018 RNA degradation (5). The DERs and DEDs which were down-regulated in TM/PTM were enriched into only 3 GO terms involving DEGs of no more than 5, and no KEGG pathway (Fig. 4).

**Figure 5:**
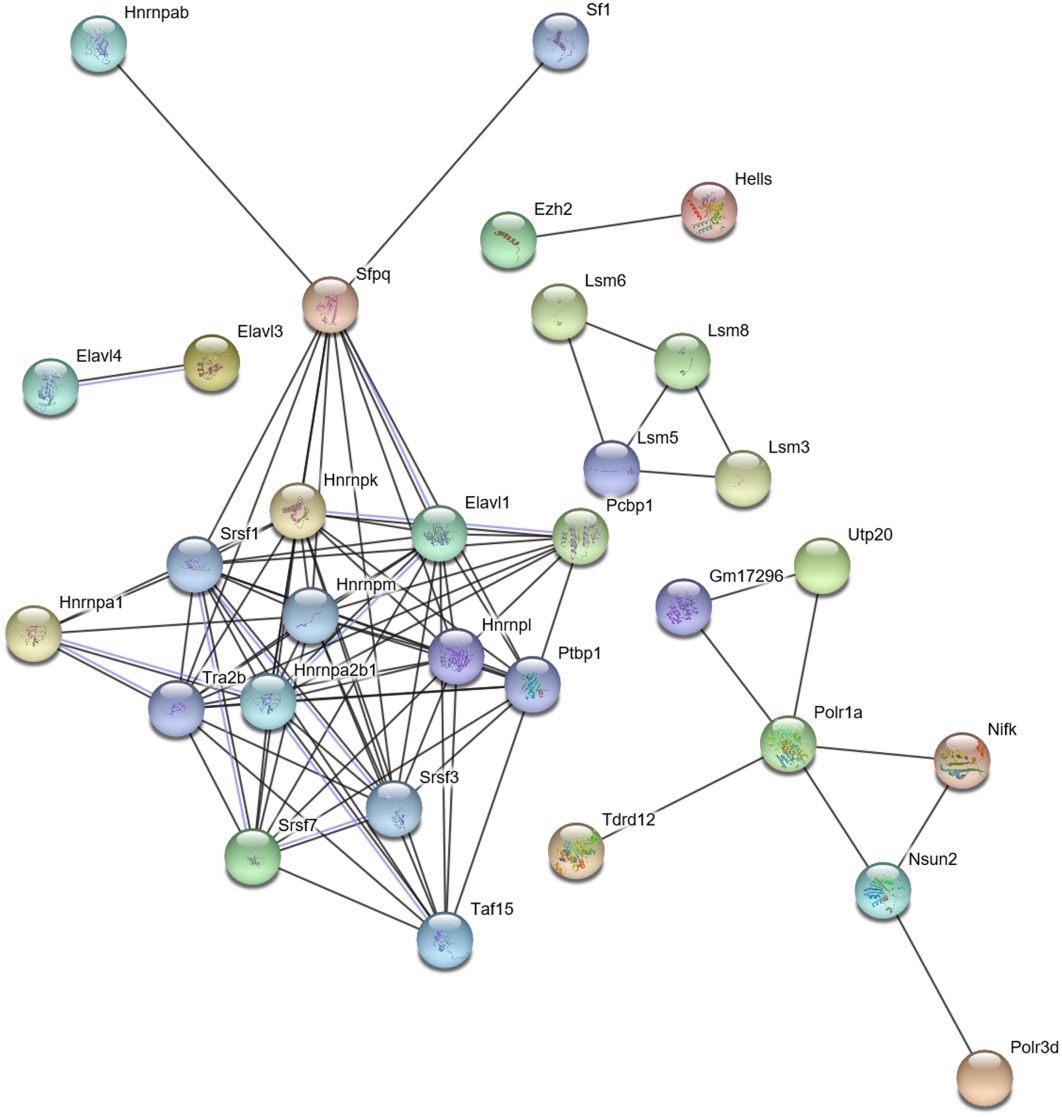
Interaction of DERs- and DEDs-encoded proteins in TM/PM. DERs, differentially expressed RNA binding protein genes; DEDs, differentially expressed genes relevant to DERs; PM, physiological microenvironment; TM, tumor microenvironment.

**Figure 6:**
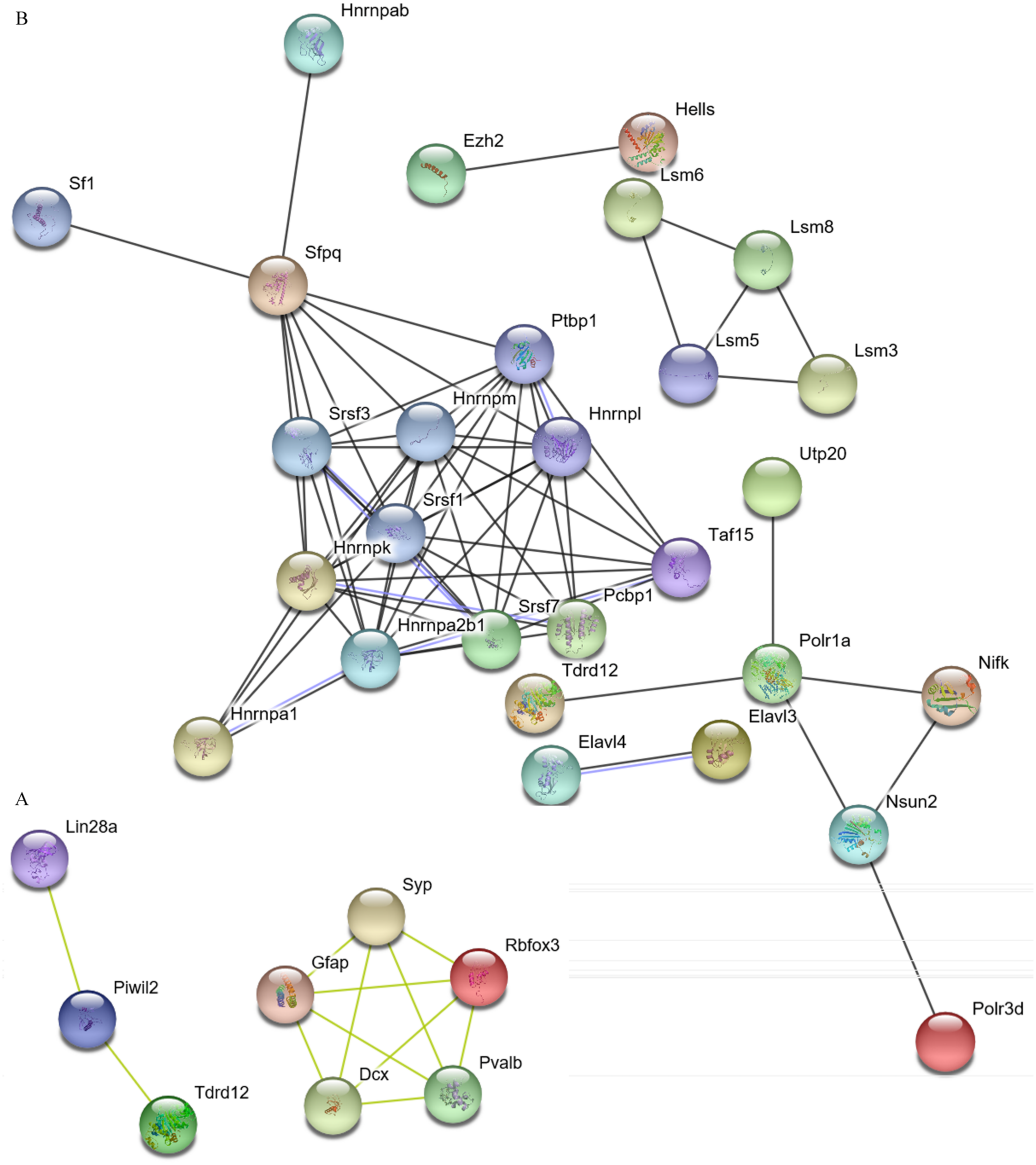
Interaction of DERs- and DEDs-encoded proteins with expression upregulation in PTM/PM and in TM/PTM. DERs, differentially expressed RNA binding protein genes; DEDs, differentially expressed genes relevant to DERs; PM, physiological microenvironment; PTM, pre-tumor microenvironment; TM, tumor microenvironment.

The interaction between the DERs- and DEDs-encoded proteins also indicated the correlation of those gene functions (Figs. 5 and 6).

### Correlation of AS and SNV/INDEL with the abnormal expression of DERs and DEDs

**Figure 7:**
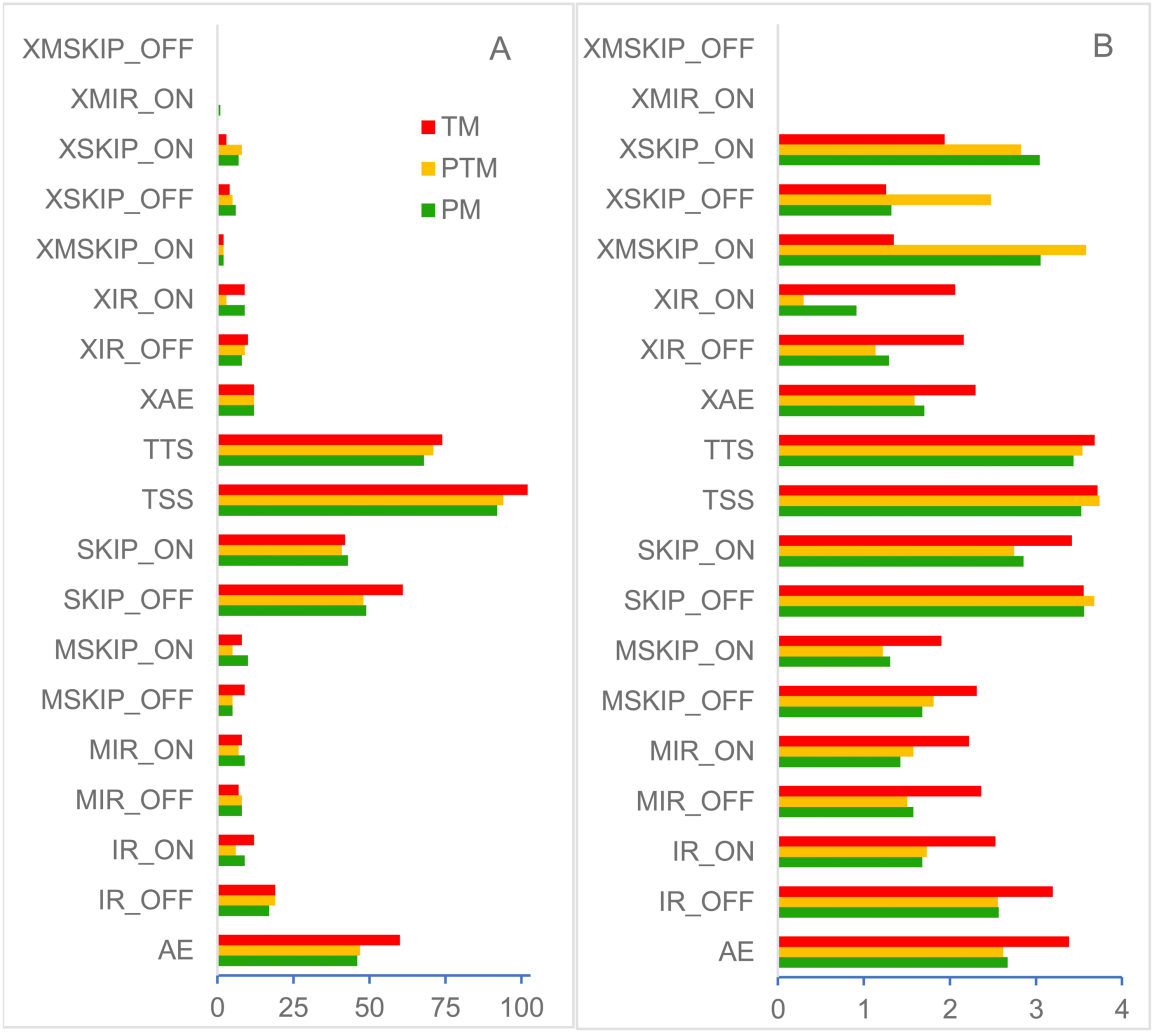
The AS of DERs and DEDs in the PM, PTM, and TM, respectively. (A) Site number pertaining to diversity AS; (B) Base 10 logarithmic value of cumulative RPKM quantity pertaining to diversity AS.

AS analysis result showed that there were 18, 19, and 19 types of AS in the PM, PTM, and TM, respectively, which had different cumulative RPKM quantity in various DERs and DEDs (Fig. 7). Especially, the occurrence frequency of most AS was positively correlated with the expression levels of DERs and DEDs (Table 2). The results of SNV and INDEL analysis showed that the occurrence frequencies of SNV and INDEL had not obvious correlation with the expression levels of DERs and DEDs in the PM, PTM, and TM.

**Table 2:** The relationship of AS frequency with the expression of DERs and DEDs.

Official gene ID	TM/PM	TM/PTM	Official gene ID	TM/PM	TM/PTM	Official gene ID	TM/PM	TM/PTM
POLR3D	+	+	ZFR		+	TIAL1	+	
KLF4	+	+	MYC	+	+	WWC2	+	+
HNRNPA2B1	+	+	TRA2B	+		LSM6	+	+
CELF1	+		CDC5L	+	+	LSM4		+
PVALB	+	+	PJA2	+	+	LSM3	+	+
PTBP1	+	+	SRSF7	+	+	ZCCHC11		+
SRP14		+	NELFE	+		STAU1		+
HNRNPL	+	+	POU5F1	+	+	ELAVL1	+	
SUZ12	+	+	SF1	+	+	LSM8	+	+
SRSF1	+	+	HELLS	+	+	HNRNPA1	+	+
HNRNPAB	+	+	CD151	+	+	IGF2		+
TAF15	+	+	NIFK	+	+	POLR1A	+	+
HNRNPK	+	+	SNRPB	+	+	HNRNPC	+	+
NSUN2	+	+	EZH2	+	+	SRSF3	+	+
PSPC1	+	+	YBX3	+	+	LSM5	+	+
						HTATSF1	−	−

**Notes.**

Note + Positive correlation − Negative correlation

## Discussion

RBPs are closely related to the functions of RNA in the cancer (Jeng et al., 2008; Hopkins et al., 2016; Dang et al., 2017). For example, LARP1, an RNA binding protein in ovarian cancer, is a post-transcriptional regulator of survival and tumorigenesis (Hopkins et al., 2016); RNA binding proteins SRSF1 and SRSF9 can promote Wnt signaling-mediated tumorigenesis by enhancing β-catenin biosynthesis (Fu et al., 2013).

In this study, we downloaded RBP genes and RBP-associated genes in *mus musculus* from the nucleotide database in NCBI and referring to data in STRING web to construct the blast genetic library. The DEGs from the RNA-seq data were then mapped into the library to picked out the DERs and DEDs. The filtered DEGs were undergone the function analysis. This analysis can more enrich and focus on the biological functions of RBPs-encoding genes because excessive unrelated genes can interfere with functional analysis of specific genes. Our results on analysis of those filtered DERs and DEDs showed that they were efficiently enriched into the biological functions on protein synthesis and relevant gene expression regulation in the PTM and TM of HCC.

In the process from the PM to the TM, there appeared 87 DERs and DEDs. Those abnormally expressed genes were widely involved in various BPs, CCs, MFs, and signaling pathways. Those functions mainly focused on the regulation of gene expression from the level of DNA and chromatin to transcription to RNA splicing and gene silencing by RNA to translation and RNA degradation. The findings suggested that the HCC were associated with the abnormal expression of RBPs-encoding genes and the relevant genes in the TM. The abnormal expression of DERs and DEDs in the HCC might affect the functions of specific RNAs by binding relevant RNAs. For example, they might directly or indirectly bind to the chromatin and/or to DNA, resulting in the activation or silencing of specific gene expression. They also might constitute spliceosome to abnormally participate in the splicing of mRNA, regulation of genes expression, causing abnormalities in protein synthesis. Other examples were the binding of miRNA or piRNA to cause the degradation of relevant RNA and result in functional disorder of their target molecules downstream of the ncRNA (Viswanathan, Daley & Gregory, 2008; Dong et al., 2019; Li et al., 2019d). They could also bind the mRNA or ribonucleoproteins to block or promote the translation of proteins (Seetharaman et al., 2016; Fei et al., 2017). Those alterations might be beneficial to the survival, migration and dwelling of liver cancer cells during the cancerization of PM (Fu et al., 2013; Bahrawy et al., 2016; Dang et al., 2017).

During the cancerization, the PTM was a transitional phase of the PM processing to the TM. In the previous study, we found a lot of DEGs in PTM/PM in the HCC (Li et al., 2019c). In the present study, we also found 18 RBPs-associated DEGs, including 4 DERs and 14 DEDs. The result indicated that both the DERs and the DEDs might be involved in the construction of PTM. Further analysis showed that, before the onset of a secondary tumor in the HCC model mice, those genes were involved in 3 GOs terms, gene silencing by RNA, nucleic acid binding, and RNA binding. The gene silencing by RNA refers to a process in which RNA silences expression of target genes. In our results, 2 DEDs, PIWIL2 (piwi-like RNA-mediated gene silencing 2) and TDRD12, and 1 DERs, LIN28A, were involved in the mRNA binding, and the slicing-triggered biogenesis and loading of MIWI2 piRNA. Those 3 genes participated in the construction of PET complex. PIWIL2 can directly bind piRNA and regulate its function. Piwil2 widely expresses in tumors and various malignancies, acting as an oncogene (Qu et al., 2015; Li & Qian, 2017). TDRD12 is essential for secondary piRNA biogenesis in mice (Pandey et al., 2013). LIN28 can promote proliferation and invasiveness of cancer cells for formation of tumor. It functions as a negative regulator of miRNA biogenesis. LIN28 can also selectively block the processing of pri-let-7 miRNAs and miRNA-mediated differentiation in stem cells in certain cancers (Viswanathan, Daley & Gregory, 2008). Among the genes involved in the mRNA binding, RBFOX3 is also a regulator in the process from pri-miRNA to pre-miRNA (Kim et al., 2014) and could promote tumor growth and progression via hTERT signaling in the HCC (Liu et al., 2017). In our study, the expression of PIWIL2, TDRD12, LIN28A, and RBFOX3 were all upregulated in PTM/PM. Those genes could inhibit apoptosis and promote proliferation of cells by the inhibition of secondary piRNA and miRNA biogenesis or binding piRNA and miRNA as an oncogene or collaborators (Viswanathan, Daley & Gregory, 2008; Pandey et al., 2013; Li & Qian, 2017). Therefore, the finding suggested that those genes were involved in construction of PTM to facilitate to the survival of cancer cells that will or are invading. Our results showed that DERs and DEDs not only participated in miRNA-related gene silencing, but also in piRNA-related gene silencing. miRNA can inhibit expression of its target gene and play various roles in biogenesis and metastasis of liver cancer. A piRNA is a large class of ncRNA first discovered in germ cells (Dehghani Amirabad et al., 2018). A piRNA plays important roles in reproduction, carcinogenesis, etc., through interacting with PIWI proteins (Yuan & Zhao, 2017). Therefore, upregulating RBPs-miRNA/piRNA axis might be a key cause for biogenesis of a PTM even a secondary tumor in a site far from the primary. In addition, ELAVL2 were involved spliceosome in KEGG pathway, ribonucleoprotein complex in molecular function and regulation of gene expression, regulation of RNA metabolic process, positive regulation of translation in BP through the interaction with its DEDs, such as Cdc5l, Hnrnpc, Hnrnpk, Pcbp1, Srsf1, Tra2b. Therefore, the expression upregulation of ELAVL2 might result in abnormality in splicing and maturation of mRNA and regulation of gene expression play important role in esophageal squamous cell carcinoma (Zhao et al., 2019). LIN28A and its DEDs, Pou5f1, Sox2, Trim71, Zcchc11, Zcchc6, were involved in miRNA binding in MF, production of miRNAs involved in gene silencing by miRNA, pre-miRNA processing in BP, discussed above. Our results suggested that RBPs might be involved in the development and metastasis before a second tumor appearance by affecting the function of related miRNA and piRNA, and mRNA splicing.

Following the PTM, the TM featured 23 DERs and 64 DEDs. They were also involved in all 3 physiological functions appeared in the stage from the PM to the PTM. However, more DERs and DEDs were involved in those physiological functions. For example, the BP “gene silencing by RNA” enriched 3 upregulated DEGs in PTM/PM, but 6 upregulated and 3 downregulated DEGs in TM/PTM; the MF “RNA binding” enriched 6 upregulated DEGs in PTM/PM, but 45 upregulated and 5 downregulated DEGs in TM/PTM; the MF “nucleic acid binding” enriched 6 upregulated DEGs in the PTM/PM, but 34 in the TM/PTM. Furthermore, the DERs in the TM also caused a lot of new physiological abnormalities. For example, the BPs, regulation of transcription, DNA-templated; mRNA splicing, via spliceosome; regulation of translation, etc., presented upregulated in TM/PTM. The relevant genes such as MALAT1, LIN28B, HMGA2, TDRD12, CELF4, PSPC1, SRSF1, SP1 etc. also are involved in the metastasis of cancer (Cui et al., 2019; Li et al., 2019c; Li et al., 2019b). The MFs, chromatin binding, core promoter binding, poly(A) RNA binding, and so on, associated genes, e.g., TRIM71, PCBP1, NIFK, CELF4, PSPC1, SP1, POLR3D, were also upregulated. The CCs, protein-DNA complex, spliceosomal complex, intracellular ribonucleoprotein complex, extracellular exosome, etc., had similar tendency of expression. The relevant genes included LSM5, PCBP2, CELF4, UTP20, SP1, POLR3D, etc. The KEGG pathways, RNA degradation and spliceosome, were also upregulated and involving genes included LSM5, SRSF3, HNRNPC, PCBP1, SNRPB, CDC5L, etc. Most of genes mentioned above also are related to the carcinogenesis or metastasis of cancer. For example, the PCBP1, SRSF3, and NIFK promotes cancer progression in metastasis or carcinogenesis (Lin et al., 2016; Liu et al., 2016; Ji et al., 2017). The most important is that some biological functions associated upregulated DEGs presented more related GO terms with more upregulated DEGs in TM/PTM than those in PTM/PM. For example, there were 12 BPs pertaining to splicing. They were mRNA splicing, via spliceosome; alternative mRNA splicing, via spliceosome; spliceosomal complex assembly; regulation of alternative mRNA splicing, via spliceosome; spliceosomal snRNP assembly; mRNA splice site selection; RNA splicing; positive regulation of RNA splicing; regulation of RNA splicing; regulation of mRNA splicing, via spliceosome; negative regulation of mRNA splicing, via spliceosome; and positive regulation of mRNA splicing via spliceosome. There were 6 CCs pertaining to splicing. They were spliceosomal complex; catalytic step 2 spliceosome; spliceosomal snRNP complex; spliceosomal tri-snRNP complex; U2-type spliceosomal complex; and U2-type catalytic step 2 spliceosome. These results indicated that a large number of DERs and DEDs took part in biogenesis of a secondary tumor after the PTM appeared and promoted the metastasis of HCC.

At the stage from the PM processing to the PTM, some RBPs-encoding genes and RBPs-associated genes belonged to the DERs and DEDs. Among them, the majority were upregulated and the minority were downregulated. There were 6 DEGs, i.e., TDRD12, RBFOX3, PUS3, LIN28A, GFAP, and C3, to be upregulated in PTM/PM but downregulated in TM/PTM. The TDRD12 and LIN28A together with TDRKH which also was upregulated in PTM/PM but downregulated in TM/PTM were enriched into 1 MF, GO:0050731 ∼positive regulation of peptidyl-tyrosine phosphorylation. Another one, PVALB, went on being upregulated at subsequent process, indicating its importance during the cancerization (Mendes et al., 2016; Zhong et al., 2018). The results demonstrated that those DEGs might have different physiological significance in the transitions from the PM to the PTM and from the PTM to the TM in biogenesis of a secondary tumor.

However, at the stage from the PTM to the TM, most RBPs-encoding genes and RBPs-associated genes were upregulated and involved in the regulation of scale gene expression. There were 13 DERs and DEDs, such as PVALB, PCBP2, IGF2, STAU1, TIAL1, SNRPB, and so on, were enriched into 1 CC, extracellular exosome. The exosome is a multivesicular body with miRNA, piRNA, lncRNA, mRNA, proteins, and liquid, with a diameter of about 40–120 nm. Exosomes play an important role in biological functions, such as signal transduction and material transport, especially in remote signaling and material transport (Hessvik & Llorente, 2018). The upregulation of those DERs and DEDs involving exosome suggested that the signal transport and material transport were inter- and intra-cellular enhanced and the exosome might play an important role during the biogenesis of a secondary tumor.

## Conclusion

In this study, we explore the biogenesis mechanism of a secondary tumor in the PM based on the functions of RBPs-encoding genes using the technology of RNA-seq and bioinformatics. The results showed that 18 DERs and DEDs were identified in the PTM/PM, 87 in TM/PTM, and 87 in the TM/PM. Those DEGs participated in the regulation of gene expression at the levels of DNA and chromatin, transcription, RNA splicing, gene silencing by RNA, and translation. The results indicated that during the biogenesis of a secondary tumor in the mouse model of HCC, the healthy PM were firstly transformed into the PTM to meet cancer cells by expression upregulation of some DERs and DEDs, which blocked the normal physiological functions of mRNA, miRNA, and piRNA by binding those RNAs when it encountered the invasion of cancer cells. However, at the stage of the PTM processing to the TM, more DERs and DEDs were upregulated and involved in the regulation of chromatin conformation, gene activation and silencing, splicing and degradation of mRNA, biogenesis of piRNA and miRNA, ribosome assemble, and translation of proteins. The abnormality of those functions in the organic microenvironments initiated the biogenesis of a secondary tumor and metastasis of HCC during the continuous counterwork of body with the cancer cells. The results demonstrated that the genes encoding RBPs and their relevant proteins were involved in the transformation from the PM to the PTM and then constructing the TM by regulating the protein synthesis. This regulation included during whole process of biological genetic information transmission based on the genetic central dogma from chromatin conformation to gene activation and silencing to splicing and degradation of mRNA to ribosome assemble to translation of proteins. The abnormality of those functions in the organic microenvironments initiated the biogenesis of a secondary tumor and promoted the metastasis of HCC when the PM encountered the invasion of cancer cells. The results also suggested that some molecules among those DERs and DEDs in the present study might be used as indicators of early location of second tumor in the development and metastasis of HCC. It needs to be further studied.

##  Supplemental Information

10.7717/peerj.8680/supp-1 Table S1 The functions of DERs and DEDs in HCC

## Additional Information and Declarations

### Data Availability

The following information was supplied regarding data availability: The RNA-seq are available at NCBI SRA: SRR8156139, SRR8156138, SRR8156137, SRR8157215, SRR8157216, and SRR8157217.
